# Genomewide comparison and novel ncRNAs of Aquificales

**DOI:** 10.1186/1471-2164-15-522

**Published:** 2014-06-25

**Authors:** Marcus Lechner, Astrid I Nickel, Stefanie Wehner, Konstantin Riege, Nicolas Wieseke, Benedikt M Beckmann, Roland K Hartmann, Manja Marz

**Affiliations:** 1 Institut für Pharmazeutische Chemie, Philipps-Universität Marburg, Marbacher Weg 6, 35032 Marburg, Germany; 2 Faculty of Mathematics and Computer Science, Friedrich-Schiller-University Jena, Leutragraben 1, 07743 Jena, Germany; 3 Faculty of Mathematics and Informatics, University of Leipzig, Augustusplatz 10, 04109 Leipzig, Germany; 4 IRI for the Life Sciences, Molecular Infection Biology, Humboldt University Berlin, Philippstr. 13, 10115 Berlin, Germany

**Keywords:** *Aquificales*, Thermophiles, ncRNA, *Aquificaceae*, *Desulfurobacteriaceae*, *Hydrogenothermaceae*

## Abstract

**Background:**

The *Aquificales* are a diverse group of thermophilic bacteria that thrive in terrestrial and marine hydrothermal environments. They can be divided into the families *Aquificaceae*, *Desulfurobacteriaceae* and *Hydrogenothermaceae*. Although eleven fully sequenced and assembled genomes are available, only little is known about this taxonomic order in terms of RNA metabolism.

**Results:**

In this work, we compare the available genomes, extend their protein annotation, identify regulatory sequences, annotate non-coding RNAs (ncRNAs) of known function, predict novel ncRNA candidates, show idiosyncrasies of the genetic decoding machinery, present two different types of transfer-messenger RNAs and variations of the CRISPR systems. Furthermore, we performed a phylogenetic analysis of the *Aquificales* based on entire genome sequences, and extended this by a classification among all bacteria using 16S rRNA sequences and a set of orthologous proteins.

Combining several *in silico* features (e.g. conserved and stable secondary structures, GC-content, comparison based on multiple genome alignments) with an *in vivo* dRNA-seq transcriptome analysis of *Aquifex aeolicus*, we predict roughly 100 novel ncRNA candidates in this bacterium.

**Conclusions:**

We have here re-analyzed the *Aquificales*, a group of bacteria thriving in extreme environments, sharing the feature of a small, compact genome with a reduced number of protein and ncRNA genes. We present several classical ncRNAs and riboswitch candidates. By combining *in silico* analysis with dRNA-seq data of *A. aeolicus* we predict nearly 100 novel ncRNA candidates.

## Background

*Aquificales* are gram-negative, non-sporulating bacteria that are thermophilic to hyperthermophilic [1,2], living in terrestrial and marine hot springs. They are autotrophs that primarily fix carbon by the tricarboxylic acid (TCA) cycle [3-5]. The hyperthermophile *A. aeolicus*, living under extreme temperatures of up to 95°C, has been proposed to have adopted 10% of its protein-coding genes by horizontal gene transfer [6,7] from Archaea. Accumulation of all the special properties of thermophiles (also referred to as accumulation profiles [8]) are rarely understood. Special protein-protective mechanisms have been analyzed [9,10], but we are far away from a comprehensive understanding of the molecular biology of extremophilic bacteria. Beyond idiosyncratic features of *Aquificales* genomes, our interest focussed on their transcriptomes. Experimentally, we performed a deep sequencing analysis on the model hyperthermophile *A. aeolicus* with the primary goal of identifying novel ncRNAs candidates. NcRNAs are known to have various functions in all domains of life. Apart from their general importance as gene expression regulators [11-13], ncRNAs are involved in processing [14] and translation [15] of other genes, in defending genomes from viral invasion [16], in shaping and maintenance of bacterial chromosome architecture [17], and they can even be multifunctional [18,19]. According to 16S rRNA analysis, the *Aquificales* constitute the most deeply rooted bacterial group [20]. However, protein-based phylogenetic reconstructions are not in line with this model [21-26].

We compared the genomes of the three *Aquificales* families, i.e. *Aquificaceae*, *Hydrogenothermaceae* and *Desulfurobacteriaceae*. We have extended the protein annotation of the mentioned *Aquificales* and reconstructed the phylogenetic position of these species based on 16S rRNAs as well as on a set of orthologous proteins. Moreover, we have identified ncRNAs based on known homologs and present a complete set of novel ncRNA candidates based on sequence analyses and deep sequencing data obtained for *A. aeolicus*. For selected ncRNA loci, we provide independent experimental evidence for their expression.

## Methods

### Genomes

We analyzed the genomes of the following species split into their respective families:

– *Aquificaceae*: *Aquifex aeolicus* VF5 (AAE), *Hydrogenivirga sp.* 128-5-R1-1 (HVI), *Hydrogenobacter thermophilus* TK-6 (HTH), *Thermocrinis ruber* (TRU), *Thermocrinis albus* DSM 14484 (TAL), *Hydrogenobaculum sp.* Y04AAS1 (HBA),

– *Hydrogenothermaceae*: *Sulfurihydrogenibium sp.* YO3AOP1 (SSP), *Sulfurihydrogenibium azorense* Az-Fu1 (SAZ), *Persephonella marina* EX-H1 (PMA), and

– *Desulfurobacteriaceae*: *Desulfobacterium thermolithotrophum* DSM 11699 (DTH), and *Thermovibrio ammonificans* HB-1 (TAM).

**Figure 1:**
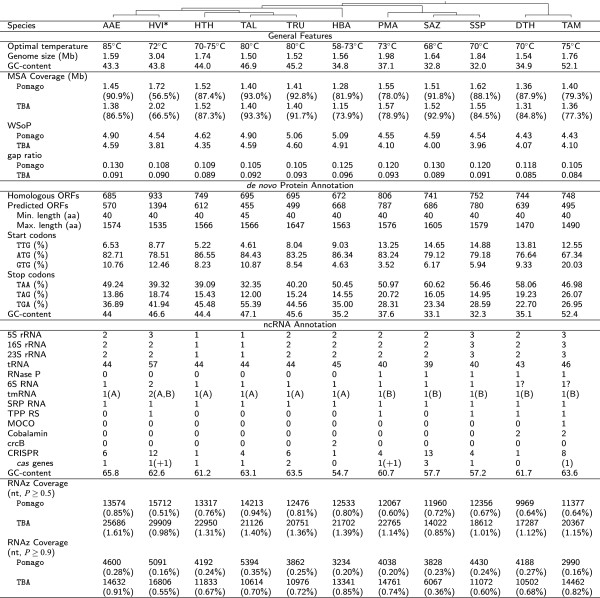
**General genome features of the** ***Aquificales*.** The genome size is given as the total number of nucleotides in the assembly. Multiple sequence alignments (MSA) were performed by Pomago and TBA. RNAz was applied to the Pomago- and TBA-derived MSAs. *De novo* protein annotation is based on statistics from BacProt, neglecting previously reported proteins for *Aquificales*. Annotation of ncRNAs shows the statistics for identified ncRNAs of known function. Details of CRISPR cassettes, number of repeats and associated proteins can be found in Figure 9 and in the Supplemental Material. TmRNAs are classified into two types (Figure 6). The phylogenetic tree shown at the top of the table is based on the whole genome as well as 16S rRNA analysis of the 11 *Aquificales* species. It reproduces the results presented in [30-32]. For further information, see Supplemental Material. AAE – *A. aeolicus*, HVI – *Hydrogenivirga sp.*, HTH – *H. thermophilus*, HBA – *Hydrogenobaculum sp.*, TAL – *T. albus*, TRU – *T. ruber*, PMA – *P. marina*, SAZ – *S. azorense*, SSP – *Sulfurihydrogenibium sp.*, DTH – *D. thermolithotrophum*, TAM – *T. ammonificans*, RS – Riboswitch, WSoP – Weighted sum-of-pairs score [29], * denotes the *Hydrogenivirga sp.* genome of unfinished assembly.

Accession numbers and sources of genomes are listed in the electronic Supplemental Material http://www.rna.uni-jena.de/supplements/aquificales/index.html. Whole-genome alignments were constructed using Pomago (v.1.0) [27] and TBA (v.11.2) (threaded blockset aligner) [28] with default parameters. Pomago alignments were computed separately for each species as reference. The TBA alignment was projected to each of the reference genomes. Coverage, alignment quality (Weighted sum-of-pairs score – WSoP [29]) and gap ratio are given in Figure 1.

### Extension of protein annotation

We used BacProt (publication in progress, see [33] for details) to complement the present annotation of protein-coding genes for each *Aquificales* genome. It uses a database of groups of orthologous protein-coding genes present in most bacteria [34]. Matches in the genome of interest are annotated, and species-specific features like codon usage, Shine-Dalgarno sequences, Pribnow box motifs and Rho-independent terminators are used to predict additional protein-coding genes. To actually achieve a *de novo* annotation, we excluded all *Aquificales* genes from the reference database. Alternative start codons like ATT and CTG were considered as well [35-37]. Re-annotated and previously annotated proteins (genomic positions and sequences) and statistics (mono-/di-nucleotide distribution, position and occurrence of Shine-Dalgarno sequence motifs and Pribnow boxes) for each species are provided in the Supplemental Material.

### Annotation of ncRNAs by homology

We used GORAP (v.1.0, publication in progress) to annotate ncRNAs in the following manner: transfer-RNAs (tRNAs) were detected by tRNAscan-SE (v.1.3.1) [38] with the option -*B* for bacteria. Split tRNAs were searched using SPLITS (v.1.1) [39]. By applying ARAGORN (v.1.2), we searched for tRNAs containing introns [40]. Searches for RNase P RNA were conducted with Bcheck (v.1.0) [41]. For the detection of putative CRISPR loci, crt (v1.2) [42] and CRISPRFinder[43] were used. We searched for *cas* protein genes by blast (v.2.2.26, E-value ≤10^-4^) [44] based on known *cas* genes (downloaded from UniProt (downloaded Jan. 2013) [45]).

To find further ncRNAs, we used blast and Infernal (v.1.1rc2) [46]. Seed sequences from the Rfam (v.11.0) database [47] and European Ribosomal RNA Database[48] were used as query with an E-value ≤0.001 for blast and the Rfam-provided family specific noise cutoff^a^ for Infernal.

NcRNAs expected to escape from detection (e.g. 6S RNA) were searched in a second step with rnabob[49] for short motif search in combination with RNAsubopt, RNAduplex, RNAcofold, RNAalifold and RNAup from the RNA Vienna Package (v.2.0) [50-53]. For verification, we aligned candidates with ClustalW (v.2.0.10) [54] or Locarnate (v.1.7.7.1) [55]. Stockholm alignments were adjusted by hand in the Emacs Ralee mode[56].

Resulting Stockholm alignments are supplied in the Supplemental Material in the General Feature Format (gff) as well as in Fasta (fa) and Stockholm (stk) formats.

### Phylogenetic reconstruction

Protein-based phylogeny was performed based on the official NCBI[57] annotations for 42 bacteria shown in the Supplemental Material. In addition to eleven *Aquificales* species, we included two Archaea as outgroup and a wide phylogenetic range of 29 bacterial species representing all bacterial clades.

Protein sequences were clustered using Proteinortho[34] in the blastp+-mode, thus performing a pairwise all-against-all comparison of sequences from different species to derive orthologous relationships. Whenever an orthologous group did not have a member in a certain species, we applied tblastn to the respective genome to complement for potentially incomplete annotations. The highest scoring alignment to an ORF above a fairly high E-value ≤ 10^-20^ was added to the initial protein annotation. Finally, Proteinortho was applied again using the expanded annotation.

For a high resolution phylogeny within the *Aquificales*, we created a whole genome alignment using Pomago. The alignment was analyzed using RAxML (v.7.4.2) [58] with a GAMMA model of rate heterogeneity with an estimate on the proportion of invariable sites and 100 rapid bootstraps.

In an additional phylogenetic analysis we used single-copy orthologous proteins present in at least 50% of all species in the set (189 groups in 42 species). Each protein group was aligned separately using dialign-tx[59]. Both ends of the group’s alignments were cropped to remove leading and tailing gaps. The remaining sequences were concatenated resulting in a 57,260 aa long alignment and applied to RAxML using the LG substitution model [60] as well as the GAMMA model of rate heterogeneity with 100 rapid bootstraps.

The 16S rRNA-based phylogeny was computed with Mafft (v.7.017) [61] using the L-INS-i method with 1000 iterations. We used different approaches: (1) Neighbor Joining with the Kimura correction model [62] (1000 bootstraps), (2) Bayesian inference with MrBayes (v.3.1.2) [63] with default parameters, (3) Maximum likelihood with RAxML (v.7.2.8) [64] (200 bootstraps) with the base substitution models (3a) GTRGAMMA (most accurate, 1000 steps) and (3b) GTRCAT for the bootstrapping phase. For all previously mentioned methods the Archaea *Methanobacterium sp.* AL-21 and *Archaeoglobus fulgidus* were used as outgroup. As state of the art, we have estimated a tree with (4) Sate (v.2.2.5) [65] (200 iterations). Related sequences were aligned with Mafft and subsequently merged by Muscle (v.3.7) [66]. The tree was computed using RAxML.

### dRNA-seq of *A. aeolicus* total cellular RNA

Transcriptome analysis of *A. aeolicus* was based on cDNA libraries from a differential deep sequencing approach (dRNA-seq) [67,68]. *A. aeolicus* cells, provided by M. Thomm and R. Huber (Regensburg, Germany), were grown for 1 day (late exponential phase) and harvested as described [69]. For preparation of total cellular RNA, we used the hot phenol method [70]: cell pellets were resuspended in extraction buffer (10 mM sodium acetate pH 4.8, 150 mM sucrose) and incubated for 10 min at room temperature with 0.1 volumes of lysozyme (20 mg/ml, Roth, Karlsruhe, Germany). SDS was added to a final concentration of 1% followed by vigorous vortexing. After addition of 1 volume phenol (preheated to 65°C) and vortexing, the mixture was incubated for 5 min at 65°C, then cooled on ice for 5 min, and centrifuged for 30 min at 4°C and 8200 g. Phenol extraction was repeated, followed by chloroform (1+1) extraction and ethanol precipitation. Finally, the DNA was digested with 10 U Turbo DNase (Ambion, Austin, USA) for 30 min at 37°C, followed by addition of another 10 U DNase and incubation for another 30 min at 37°C. Subsequently, the RNA was subjected to phenol/chloroform extraction and ethanol precipitation. After redissolving the RNA in double-distilled water, its concentration was determined by UV spectroscopy. Before cDNA library construction, the RNA was split into two fractions; one fraction was treated with Terminator 5’ P-dependent exonuclease (Epicentre, Madison, USA) for depletion of transcripts carrying a 5’-monophosphate. Both fractions were treated with Tobacco Acid Phosphatase (TAP) before 5’-linker ligation, poly(A) tailing and conversion into cDNA (vertis Biotechnologie AG, Freising, Germany). The cDNA libraries were then sequenced on a Roche FLX sequencer and resulted in the (-)-library with 25,816 reads and the (+)-library (33,697 reads) containing the enriched primary transcripts.

### Detection of novel ncRNAs

We used the IGB (Integrated Genome Browser) [71] to visualize the following features of *A. aeolicus*: (1) nucleotide sequence; (2) local GC-content (for each nucleotide 15 nt on both sides were included for the calculation of GC-content); (3) protein genes annotated by NCBI[72] and BacProt; (4) locally stable secondary structures: calculation was performed with RNALfold with options -*d*2 and -*L*120 for both strands with a maximum base-pair span of 120 nucleotides. Sequences with local structures of fewer than 50 nt were discarded. For the prediction of thermodynamically stable RNA structures, each sequence was shuffled 1000 times while preserving the dinucleotide frequencies; to classify extraordinarily stable RNA secondary structures, we chose to use a Z-score cutoff of -3.0 (∼ top 5% of stable structures); (5) conserved regions among the *Aquificales*: with default parameters of TBA and Pomago we aligned 11 genomes; the TBA alignment was projected to each of the reference genomes; coverage, WSoP and gap ratio are given in Figure 1; (6) novel ncRNAs: novel ncRNA candidates were predicted using RNAz. We used rnazWindow.pl –min-seqs=4 and RNAz -n -b -p 0.5 on the alignments of Pomago and TBA. As rnazWindow.pl assumes lower case nucleotides to be masked, the alignments were converted to upper case letters beforehand; (7) dRNA-seq: cDNA libraries were mapped with segemehl (v.0.0.9.3) [73] applying the parameters -m 12 -D 1 -e 2 -p 4 -X 8 -A 90 -E 5.0.

### Northern blot experiments

#### Total RNA preparation

Total RNA was prepared from cell pellets using the hot phenol method as described [74].

#### Positive and negative controls

The positive and the negative controls for the Northern blot experiments were synthesized by *in vitro* transcription using the "TranscriptAid T7 High Yield Transcription Kit" (Thermo Scientific, Germany), according to the protocol supplied by the manufacturer. PCR products generated with the "Long PCR Enzyme Mix" (Thermo Scientific) served as templates for *in vitro* transcription. As positive controls for the antisense tRNA blots, chemically synthesized RNA oligonucleotides from "Integrated DNA Technologies" (IDT, Belgium) were used (for sequences, see Supplemental Material). RNA oligonucleotides were 5’-phosphorylated before gel electrophoresis. The *in vitro* transcribed full-length sense tRNAs (generated from PCR products) were used as negative controls for the Northern blots of antisense tRNAs.

#### Digoxigenin and LNA probes

For the Northern blot detection internally digoxigenin-labeled probes were transcribed using the DIG RNA Labeling Mix (Roche Diagnostics, Germany) as described [74]. The antisense tRNA transcripts were detected with chemically synthesized 5’-digoxigenin-labeled DNA/LNA mixmer probes (Exiqon, Denmark; for sequences, see Supplemental Material).

#### 5’-Phosphorylation of RNA oligonucleotides

67 ng/ *μ*l RNA oligonucleotide, 2.5 mM DTT, 2 mM ATP and 10 U T4 polynucleotide kinase (T4 PNK; Thermo Scientific) were incubated in 1 × T4 PNK buffer (Thermo Scientific) in a volume of 15 *μ*l for 1 h at 37°C, followed by transfer to and storage at -20°C.

#### Electrophoresis

RNAs were separated on 8% or 10% denaturing (8 M urea) PAA gel with 1 × TBE as electrophoresis buffer [74].

#### Blotting, crosslinking, hybridization and detection

RNA blotting, hybridization (EDC crosslinking or baking at 80°C for 40 min) and immunological detection were performed as described [74], except that RNA blotting was carried out at 0.36 mA/cm ^2^ overnight. Prehybridization and hybridization were performed at 68°C (except for 50°C in the case of antisense tRNA 44) using 12 ml hybridization solution. 3.5 *μ*l of *in vitro* transcribed, internally digoxigenin-labeled probe were added for overnight hybridization. 300 pmol of chemically synthesized, 5’-digoxigenin-labeled DNA/LNA mixmer probe were used for Northern detection of antisense tRNAs. Blotted membranes were stored at room temperature.

#### *In vitro* transcripts, probes and primers

Further details on *in vitro* transcripts, probes and primers are listed in the Supplemental Material.

## Results and discussion

### Genome analysis – general observations

The genomes of the *Aquificales* range from 1.50 Mb (*T. albus*) to 1.98 Mb (*P. marina*), thus being at the lower limit of bacterial genomes ranging in size from 0.14 to 14.38 Mb with a mean of ∼ 4 Mb [75]. The current annotation file of *Hydrogenivirga sp.* contains 3.04 Mb, which is considerably larger than the genome size of the other *Aquificales*, which might be an assembly artefact as discussed later.

*Aquificales* are known to be AT-rich with a GC-content of about 43% [72,76]. In *Hydrogenobaculum sp.*, *Sulfurihydrogenibium sp.* and *S. azorense* even only one-third of the nucleotides are guanine or cytosine. For *T. ammonificans* an atypically high GC-content of more than 50% was observed.

Between 6.5% (*S. azorense*) and 28.5% (*Hydrogenobaculum sp.*) of the genomes were found to be unique to each member bacterium (Figure 1). The comparatively low coverage of *Hydrogenivirga sp.* is due to the currently assembled genome being almost twice as long as those of other *Aquificales*. 10.5% to 13.0% of the Pomago alignment, resp. 8.4% to 9.6% of the TBA alignment, consist of gaps. According to the WSoP each nucleotide from the alignment is conserved on average in slightly less than half of the other 10 species (4.43 to 5.09 out of 11 and 3.81 to 4.91 out of 11, for Pomago and TBA, respectively) indicating that the genomes diverged relatively fast. Genomic rearrangements among the *Aquificales*, underlining the diversity, can be seen in an overview of the Pomago alignment in the Supplemental Material.

### Extended annotation of proteins

We extended the original NCBI annotation of proteins of the *Aquificalesde novo* using BacProt, revealing a number of additional proteins (Table 1). Since a large fraction of proteins are hypothetical or of unknown function, we added for each species a second row which exclusively depicts those with an associated function. The annotations of NCBI and BacProt were merged to generate an extended annotation of protein genes in the *Aquificales*.

**Table 1:** Protein annotations

	**NCBI**	**BacProt**	**Equal**	**Start shifted**	**End shifted**	**NCBI** only	**BacProt** only	**Extended**
AAE	**1560**	1255	954	116	124	366	61	**1621**
	897	685	475	51	54	317	105	1002
DTH	**1513**	1383	1092	86	105	230	100	**1613**
	1115	744	561	58	74	422	51	1166
HBA	**1629**	1340	1040	119	126	344	55	**1684**
	1063	672	500	62	68	433	42	1105
HTH	**1893**	1361	1069	111	129	584	52	**1945**
	1343	749	594	62	84	603	9	1352
PMA	**2051**	1593	1286	129	122	514	56	**2107**
	1494	806	629	84	76	705	17	1511
SAZ	**1723**	1427	1190	90	99	344	48	**1771**
	1321	741	601	50	73	597	17	1338
SSP	**1722**	1532	1225	76	108	313	123	**1845**
	1145	752	573	38	70	464	71	1216
TAL	**1593**	1145	903	93	127	470	22	**1615**
	1144	691	514	59	85	486	33	1177
TAM	**1814**	1243	1014	90	99	611	40	**1854**
	1176	748	575	60	63	478	50	1226
HVI	**3808**	2327	1537	302	306	1663	182	**3990**
	1960	933	595	102	92	1171	144	2104

Annotations obtained with NCBI (first column, bold font) and those identified with BacProt (second column) lead to an extended current annotation of *Aquificales* (last column, bold font). In the second lines, hypothetical proteins were removed. Equal – proteins equally identified by BacProt and NCBI; Start/End shifted – proteins identified by BacProt and NCBI vary in length (only 5’ or 3’); NCBI/BacProt only – proteins identified only by NCBI/BacProt. All gff files are available in the Supplemental Material. Species abbreviations as in Figure 1.

We added between 0.7% of *H. thermophilus* (1352/1343) and 10.6% of *A. aeolicus* (1002/897) protein-coding genes to the NCBI annotation.

**Figure 2:**
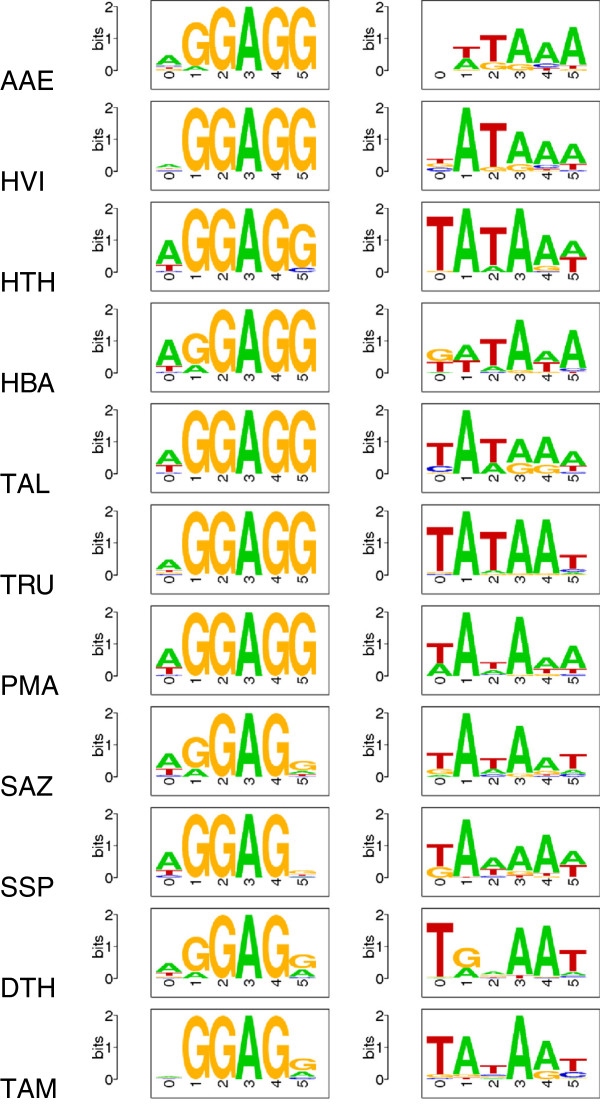
**Shine-Dalgarno sequence motifs (left) and Pribnow box (-10 box) motifs (right) in the *Aquificales*.** Details can be found in the Supplemental Material. For species abbreviations see Figure 1.

For all proteins annotated by BacProt, we extracted the Shine-Dalgarno and Pribnow box (-10 box) motifs (see Figure 2) in order to facilitate the assignment of novel *Aquificales*-specific proteins. The Shine-Dalgarno sequence is rather conserved (GGAGG, but always NGAGN). In contrast, the Pribnow box is recognizable but less conserved, indicating more sequence variations among promoters. With the appropriate covariance models we searched for species-specific novel proteins and listed them as predicted proteins in the Supplemental Material.

An overview of the codon usage of *A. aeolicus* is shown in Table 2. Complete data on all codon usage tables and mono/dinucleotide distributions are provided in the Supplemental Material. We observe a disproportionate usage of certain triplets: isoleucine is mostly (63%) encoded by AUA, tyrosine by UAC (82%) and histidine by CAC (84%). The four arginine codons with a cytosine at the first position of the triplet are rarely used, compared to the two adenine-containing triplets (9%/91%).

**Table 2:** **Codon usage of** ***A. aeolicus***

	**Codon**	**aa**	**%**	**Fraction**	**Codon**	**aa**	**%**	**Fraction**	**Codon**	**aa**	**%**	**Fraction**	**Codon**	**aa**	**%**	**Fraction**	
U	UUU	Phe (F)	2.9	0.56	UCU	Ser (S)	0.9	0.18	UAU	Tyr (Y)	0.8	0.18	UGU	Cys (C)	0.4	0.49	U
	UUC	Phe (F)	2.3	0.44	UCC	Ser (S)	1.3	0.27	UAC	Tyr (Y)	3.4	0.82	UGC	Cys (C)	0.4	0.51	C
	UUA	Leu (L)	1.7	0.16	UCA	Ser (S)	0.7	0.15	UAA	stop	0.1	0.49	UGA	stop	0.1	0.37	A
	UUG	Leu (L)	0.8	0.08	UCG	Ser (S)	0.4	0.07	UAG	stop	0	0.14	UGG	Trp (W)	0.9	1	G
C	CUU	Leu (L)	2.7	0.25	CCU	Pro (P)	1.1	0.26	CAU	His (H)	0.3	0.16	CGU	Arg (R)	0.2	0.03	U
	CUC	Leu (L)	3.1	0.3	CCC	Pro (P)	1.8	0.42	CAC	His (H)	1.3	0.84	CGC	Arg (R)	0.1	0.03	C
	CUA	Leu (L)	0.8	0.07	CCA	Pro (P)	0.6	0.14	CAA	Gln (Q)	0.7	0.35	CGA	Arg (R)	0.1	0.01	A
	CUG	Leu (L)	1.4	0.14	CCG	Pro (P)	0.7	0.18	CAG	Gln (Q)	1.3	0.65	CGG	Arg (R)	0.1	0.02	G
A	AUU	Ile (I)	1.7	0.23	ACU	Thr (T)	1	0.23	AAU	Asn (N)	1.1	0.3	AGU	Ser (S)	0.8	0.16	U
	AUC	Ile (I)	1	0.13	ACC	Thr (T)	1.2	0.27	AAC	Asn (N)	2.5	0.7	AGC	Ser (S)	0.8	0.17	C
	AUA	Ile (I)	4.6	0.63	ACA	Thr (T)	0.9	0.21	AAA	Lys (K)	4.4	0.48	AGA	Arg (R)	1.9	0.38	A
	AUG	Met (M)	1.8	1	ACG	Thr (T)	1.2	0.29	AAG	Lys (K)	4.8	0.52	AGG	Arg (R)	2.6	0.53	G
G	GUU	Val (V)	3	0.38	GCU	Ala (A)	1.6	0.26	GAU	Asp (D)	1.6	0.37	GGU	Gly (G)	1.6	0.23	U
	GUC	Val (V)	0.9	0.11	GCC	Ala (A)	1.3	0.21	GAC	Asp (D)	2.7	0.63	GGC	Gly (G)	0.9	0.12	C
	GUA	Val (V)	2.5	0.32	GCA	Ala (A)	1.7	0.29	GAA	Glu (E)	6.2	0.65	GGA	Gly (G)	3.4	0.5	A
	GUG	Val (V)	1.5	0.19	GCG	Ala (A)	1.4	0.24	GAG	Glu (E)	3.3	0.35	GGG	Gly (G)	1	0.15	G
	U				C				A				G				

Codon usage is based on 1,255 protein-coding genes comprising 431,072 codons. Codon usage of other Aquificales can be viewed in the Supplemental Material. aa – amino acid; the fraction of a particular amino acid encoded by the respective codon is given (1 for Trp encoded by a single codon).

### Homology search and annotation of known ncRNAs

A search for ncRNA candidates with RNAz[77] predicted a relatively constant fraction of the genome to code for ncRNAs (between 0.36% for *S. azorense* and 0.91% for *A. aeolicus*). Besides the well-known and described rRNAs and tRNAs, only a handful of other wide-spread ncRNAs were detected (Figure 1).

#### rRNA operons

Most of the *Aquificales* genomes have two rRNA operons (Figure 1). *H. thermophilus* and *T. albus* appear to harbor only one operon. The genomes of *T. ammonificans* and *Sulfurihydrogenibium sp.* contain three operons, whereas *Hydrogenivirga sp.* appears to have two 16S, two 23S and three 5S rRNA genes.

#### tRNAs

With the exception of *Hydrogenivirga sp.* (see below), tRNAscan identified between 39 (*S. azorense*) and 46 tRNAs (*T. ammonificans*) per *Aquificales* species. With SPLITS and ARAGORN no split tRNAs were found.

All possible codons are utilized in the *Aquificales* (see Table 2 for *A. aeolicus*, and Supplemental Material for other *Aquificales*), but the number of tRNA genes is reduced to a minimum in contrast to reference bacteria such as *E. coli* which encodes multiple copies of many tRNA isoacceptors.

**Figure 3:**
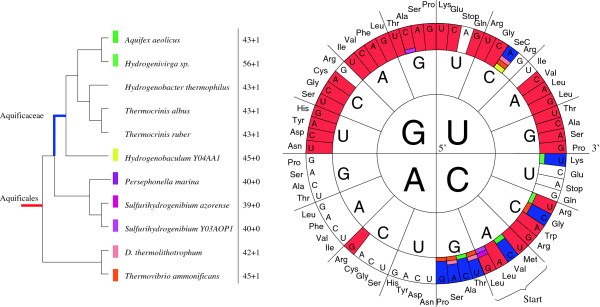
**Distribution of tRNAs in the** ***Aquificales*.** Left: The total numbers of encoded tRNAs including the absence (+0) or presence (+1) of selenocysteine tRNA (tRNA-SeC) are given. Phylogenetic tree as in Figure 1; Right: anticodons specified by the following colors: red – tRNA with this anticodon encoded in all *Aquificales*; blue – tRNA encoded in the *Aquificaceae* only. Other colors represent the absence or presence of a tRNA with this anticodon, as defined in the phylogenetic tree on the left. For example, tRNA-SeC is present in all *Aquificaceae* except for *Hydrogenobaculum sp.*, and is additionally found in the non-*Aquificaceae* species *D. thermolithotrophum* and *T. ammonificans*.

**Figure 4:**
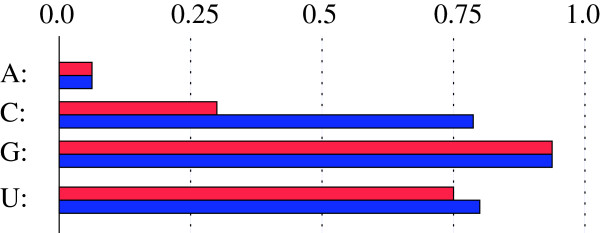
**The distribution of the 5’ (wobble) position of a tRNA anticodon in all *Aquificales * (red) versus *Aquificaceae * only (blue) is U: 0.75/0.80%, G: 0.93/0.94%, C:0.30/0.78%, A:0.06/0.06%.** Non-*Aquificaceae* show a low percentage of wobble C.

Figure 3 shows nearly no tRNA with 5’-A in the anticodon and only half of the *Aquificales* have some anticodons with 5’-C, where the non-*Aquificaceae* apparently favored the reduction of such tRNA genes (Figure 4). Important tRNA modification enzymes (TadA – tRNA adenosine deaminase and TilS – tRNA-Ile lysidine synthetase) are encoded in *Aquificales* and X-ray structures of TadA and TilS from *A. aeolicus* have been reported [78,79]. TadA converts A residues in the 5’-position of certain tRNA anticodons to inosine to expand wobble decoding, and TilS converts the 5’-C residue in the CAU anticodon of specific tRNA-Ile molecules to lysidine (2-lysyl cytidine; abbreviated as *L* or *k*^2^*C*) to decode 5’-AUA (Ile) codons instead of 5’-AUG (Met) codons [80]. Without this posttranscriptional modification, decoding of isoleucine AUA codons would be impossible [81-83]. Selenocysteine-specific tRNAs decoding 5’-UGA are present in the *Aquificaceae* (except for *Hydrogenobaculum sp.*) and in the *Desulfurobacteriaceae* (*T. ammonificans* and *D. thermolithotrophum*), but are absent from the *Hydrogenothermaceae* (*P. marina*, *S. azorense*, *Sulfurihydrogenibium sp.*; see Figure 3). The *Aquificaceae* (except *Hydrogenivirga sp.*), in contrast to the other *Aquificales* or mesophiles such as *E. coli* or *B. subtilis*, encode the lysine isoacceptor with the anticodon 5’-CUU to decode the AAG codon.

#### RNase P

The catalytic RNA subunit of the tRNA processing endoribonuclease RNase P was previously identified in *P. marina* and *S. azorense*[84]. Additionally, RNase P RNAs were easily identified here with Bcheck in *Sulfurihydrogenibium sp.*, *T. ammonificans* and *D. thermolithotrophum*. In the *Aquificaceae*, RNase P RNA candidates were neither detected with Bcheck, rnabob nor by manual *in silico* search methods using cDNA libraries of *A. aeolicus*. This is consistent with the negative results of previous searches for RNase P RNA in *A. aeolicus*[85,86].

All identified RNase P RNAs lack the P18 element, which appears to be a general feature of type A RNase P RNAs in the *Hydrogenothermaceae* and *Desulfurobacteriaceae*. The *Sulfurihydrogenibium sp.*, *T. ammonificans* and *D. thermolithotrophum* RNAs differ from their *P. marina* and *S. azorense* counterparts by a weaker L9-P1 tertiary contact (L9 5’-GYAA tetraloop docking on an A-U/G-C tandem bp instead of a G-C/G-C tandem which is a hallmark of RNase P RNAs from thermophiles [84,87]). Other differences are: (1) very short P12 stems in *T. ammonificans* and *D. thermolithotrophum*, (2) particularly weak P17 stems in *Sulfurihydrogenibium sp.* and *D. thermolithotrophum*, (3) a destabilized L8-P4 interaction, a destabilized P14 helix, but a stabilized L14-P8 interaction in *T. ammonificans*. For details, see RNase P RNA 2D structures in the Supplemental Material.

#### 6S RNA

Bacterial 6S RNAs, about 200 nt in length, form a rod-shaped secondary structure with a central bulge region flanked by largely helical arms on both sides. Their structure is thought to mimic the structure of an open DNA promoter [88,89]. 6S RNAs bind to the housekeeping RNA polymerase holoenzyme to block transcription at DNA promoters, primarily upon entry of cells into stationary growth phase. When nutrients are resupplied (including NTPs), RNA polymerase massively synthesizes transcripts (so-called product RNAs – pRNAs) on 6S RNA as template, which lead to a structural rearrangement of 6S RNA and release of RNA polymerase. Thus, 6S RNA is a fast riboregulator that makes RNA polymerase instantly available for a new exponential growth when nutrients are resupplied [68,90-93].

In *A. aeolicus* the 6S RNA was clearly identified via an experimental RNomics approach [85]. 6S RNA candidates in the other *Aquificales* were predicted computationally using the Rfam covariance model and, as expected, vary substantially in primary, but less in secondary structure. For *Hydrogenivirgia* we found two copies. Predicted 6S RNAs for *T. ammonificans* and *D. thermolithotrophum* remain candidates since they differ substantially from those of other *Aquificales*.

The RNAalifold consensus structure for the 6S RNA candidates from all other *Aquificales* analyzed here is shown in the Supplement. Individual RNAfold predictions (see Supplemental Material for details) support the notion that they are *bona fide* 6S RNAs.

**Figure 5:**
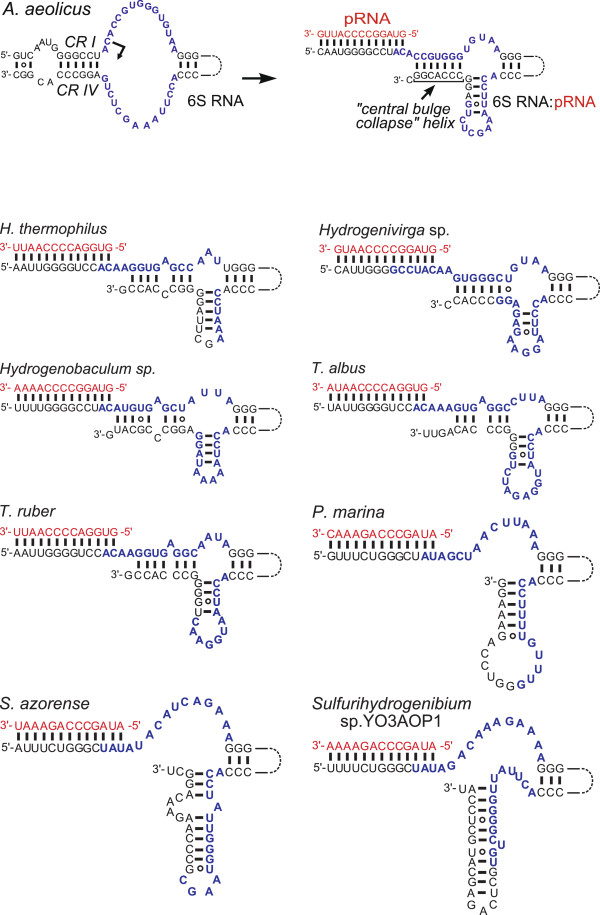
***Aquificales* 6S RNAs: predicted pRNA transcription initiation sites, and pRNA-induced structural rearrangements of 6S RNAs.** Top: *A. aeolicus* 6S RNA; this 6S RNA was experimentally verified [85] and the pRNA transcription start site identified by deep sequencing (unpublished results); nucleotides of the central bulge region are marked in blue; during pRNA transcription on 6S RNA as template, the endogenous helix is disrupted, leading to the formation of new base-pairing interactions. Here, a 6S RNA hybrid with a pRNA 13-mer (red) is shown on the right; the proposed rearranged structure of the central 6S RNA region [90] has not yet been proven experimentally. Proposed structures of the central bulge regions and their pRNA-induced rearrangements of the other eight *Aquificales* 6S RNA candidates: rearranged structures upon duplex formation with putative pRNA 13-mers; the pRNA initiation sites are proposed on the basis of resemblance to *A. aeolicus* 6S RNA. For more details, see Supplemental Material.

In the case of *A. aeolicus* 6S RNA, we proposed that formation of a "central bulge collapse" helix (Figure 5-Top, [85]) is the major component of the pRNA-induced rearrangement of this 6S RNA structure [90]. If at all, or to which extent, the adjacent hairpin structure forms in the pRNA-rearranged structure remains to be investigated. For the eight other 6S RNA candidates (Figure 5), we predicted rod-shaped structures with a destabilized central region that is not necessarily purely single-stranded (see Supplemental Material for further details). According to our proposals, pRNAs would start with a G residue in the *Aquificaceae*, whereas those of the *Hydrogenothermaceae* (*P. marina*, *S. azorense* and *Sulfurihydrogenibium sp.*) would initiate with an A residue.

#### tmRNA

**Figure 6:**
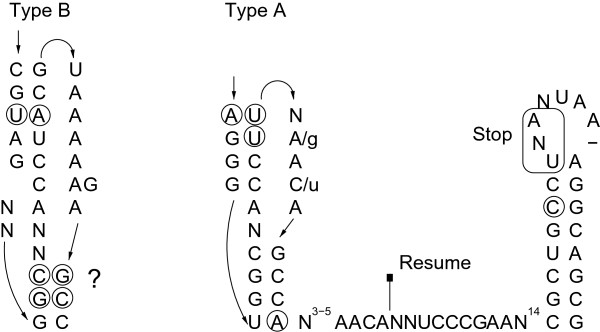
**Pseudoknot 1 (pk1) of tmRNAs type A and B.** Formation of the lower stem of pk1 is questionable. Circled nucleotides represent positions of compensatory mutations. The question mark shows an unclear interaction of two (in one case three) base pairs.

In bacteria, stalling of translating ribosomes on truncated mRNAs is rescued through action of the dual-function transfer-messenger RNAs (tmRNAs) [94,95]. The tRNA-like domain is present and highly conserved in all *Aquificales*. An architectural feature of tmRNAs is their intricate structure consisting of four pseudoknots. Interestingly, we found two different types of tmRNAs, introduced here as type A (present in the *Aquificaceae*) and B (specific to *Hydrogenothermaceae* and *Desulfurobacteriaceae*). This classification is based on the observation that the lower stem of pseudoknot 1 (pk1) involves 4-5 bp in type A tmRNAs, but only 2–3 bp in type B variants (Figure 6, Supplemental Material). Pk1 is critical for tmRNA function and binds near the ribosomal decoding site [95]. Mutational analysis of *E. coli* tmRNA revealed that mutations disrupting the upper stem of pk1 are not tolerated, whereas the outer two base pairs of the lower stem (Figure 6) can be disrupted (resulting in a 3-bp stem) without loss of function [95]. On the other hand, the tmRNA of another thermophile, *Thermotoga maritima*, has a lower pk1 stem expanded to 7 bp [96]. This raises the question if the *Aquificales* type B tmRNAs, for which only a 2-bp lower pk1 stem is predicted (*Sulfurihydrogenibium sp.*, *P. marina* and *S. azorense*), are still able to form this pseudoknot, or if the weakness or absence of this stem is compensated for by e.g. tmRNA ligand interactions that are idiosyncratic to the *Aquificales* encoding a type B tmRNA.

**Figure 7:**
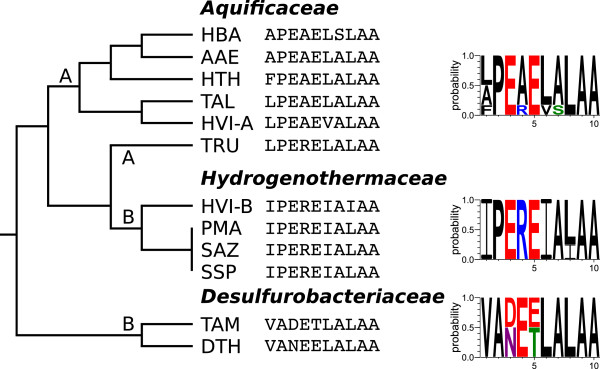
**Proteolysis tags of tmRNA types (A/B).** The encoded proteolysis tag as well as a probability logo for each family are shown. Two tmRNAs were identified in the genome assembly of *Hydrogenivirga sp*.

The messenger RNA-like regions (MLR), which are in close vicinity of pk1, encode tag preptides of 10 amino acids, with subphyla-specific signatures (Figure 7). For example, all *Aquificaceae* and *Hydrogenothermaceae* tmRNAs code for a proline at the second position, which is alanine in the *Desulfurobacteriaceae*. The genome of *Hydrogenivirga sp.* appears to encode both types of tmRNAs (type A and B). Whether this reflects a genuine tmRNA gene duplication rather than a genome contamination or assembly artefact remains to be clarified (see below).

**Figure 8:**
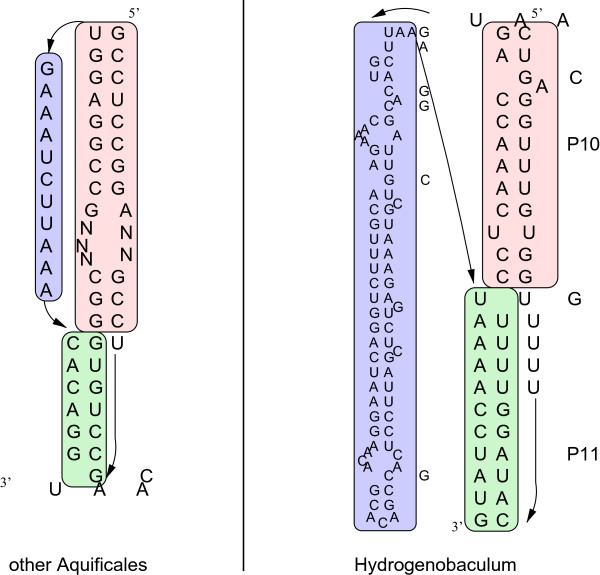
***Hydrogenobaculum sp.* has a 78-nt insertion downstream of P10, but pseudoknot 4 (pk4) is still predicted to form.** The region is shown in comparison to *T. albus* as a representative of the other *Aquificales*. Detailed figures of all tmRNAs can be viewed in the Supplemental Material.

Furthermore, *Hydrogenobaculum sp.* carries a 78-nt hairpin-like insertion in the pseudoknot 4 (pk4) region, which however is compatible with formation of pk4 (Figure 8). Such a long extension within tmRNAs has been not reported yet.

#### CRISPR system

**Figure 9:**
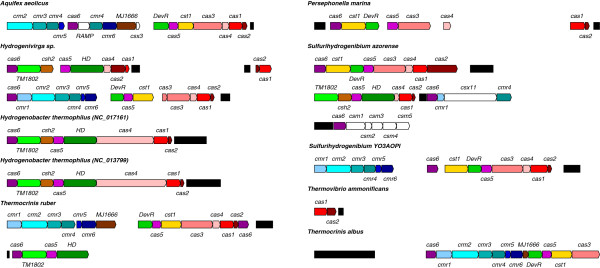
**CRISPR clusters and associated** ***cas* protein genes in the** ***Aquificales*.** Shown *cas* genes and CRISPR clusters (black filled boxes) are proportional in size. As only CRISPR clusters with associated *cas* genes are displayed in this figure, *Hydrogenobaculum sp.* and *D. thermolithotrophum* are not displayed here.

For each member of the *Aquificales* we could identify at least one locus of clustered interspaced short palindromic repeat sequences (CRISPRs), which are involved in an immunity against viruses and plasmids [97]. Although the *Aquificales* have very compact genomes, the number of identified CRISPR clusters varied from one to thirteen (Figure 1), indicating the presence of thermostable viruses in extreme environments as reported for Archaea [98]. The number of CRISPR clusters does not seem to be clade-specific. Also, the number of repeats in a cluster varies strongly. For example, in *T. albus* we found in total four CRISPR systems containing 36, 41, 57 and 63 repeats, whereas in *A. aeolicus* the five CRISPR loci only had four to five repeats. For some, but not all of the CRISPR clusters, we could detect associated *cas* genes (Figure 9). The exact numbers of detected CRISPR clusters and Cas protein cassettes can be seen in Figure 1. In this table we included only CRISPR clusters that were found by both approaches (crt and CRISPRfinder). It has to be kept in mind that the genome of *Hydrogenivirga sp.* is in an unfinished state, so it is possible that some CRISPR loci and especially associated *cas* genes escaped detection.

#### Other ncRNA

SRP RNA was found once per genome being highly conserved in sequence and structure (see Supplemental Material). Additionally, we show some riboswitch candidates: TPP, MOCO, Cobalamin and crcB (see Figure 1). The MOCO riboswitch found in *T. ammonificans* and the two crcB riboswitches identified in *Hydrogenobaculum sp.* conform well to the Rfam conservation model (see Supplemental Material). Riboswitches were only found sporadically among the *Aquificales*.

### Novel ncRNAs in *A. aeolicus*

Besides the annotation of ncRNAs with known functions, we additionally aimed to detect novel ncRNAs, as they often regulate transcription or play an important role as posttranscriptional regulators. Here we combined *in silico* analysis of the *A. aeolicus* genome and dRNA-seq data from the same organism to identify novel ncRNA candidates, some of which were subsequently analyzed by Northern blot analysis.

In the *in silico* search, small ncRNAs (sRNAs) were distinguished from proteins by the following analysis steps: (1) The GC-content of the *A. aeolicus* genome is 43%. However, the ncRNAs described above show an average GC-content of 66%. We associated each nucleotide with a local GC-value. (2) The function of small ncRNAs, e.g. 6S RNA, is often determined by their stable secondary structure. To each position in the genome, we assigned the minimum free energy of the most stable local secondary structure including this nucleotide, using RNALfold. (3) Most ncRNAs are conserved among closely related organisms. We calculated genomewide multiple sequence alignments (MSA) with TBA and Pomago of all *Aquificales* genomes, which can be viewed in the Supplemental Material. (4) Based on the MSAs we performed a novel ncRNA prediction with RNAz and displayed their probability.

**Figure 10:**
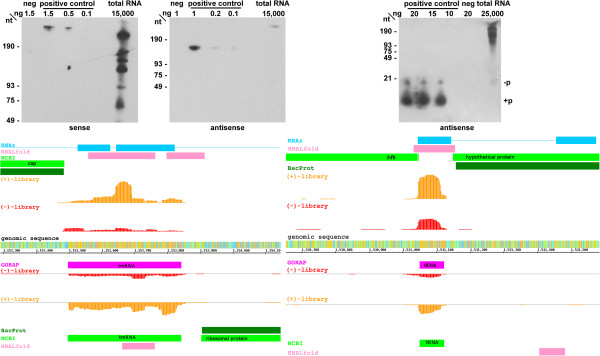
**cDNA read profiles (bottom) and Northern blots (top) of selected ncRNAs.** Read profiles left: tmRNA (350 counts); right: tRNA 44 (1000 counts); The upper half of each read profile represents the plus strand and the lower one the minus strand. Annotation by RNAz (blue), RNALfold (rose) and NCBI/BacProt (green), cDNA reads of the (+)-library (orange) and of the (-)-library (red) and ncRNA annotation by GORAP (pink); colors of genomic sequences represent nucleotides A (green), C (red), G (orange) and T (blue); counts - scale of read display adapted to the maximal number of detected reads (see Table 3). For Northern blots, see Methods; -p – with 5’-OH ; + p – with 5’ monophosphate.

**Figure 11:**
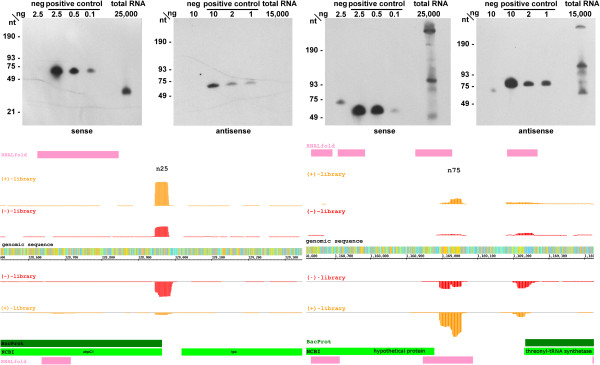
**cDNA read profiles (bottom) and Northern blots (top) of selected ncRNAs.** Read profiles left: n25 (600 counts); right: n75 (250 counts). Details as in Figure 10. For Northern blots, see Methods.

All ncRNA candidates with a minimum length of 25 nt and not overlapping protein-coding sequences, rRNA operons or tRNAs, were summarized in a full candidate table, containing all properties mentioned above (see Supplemental Material). A subset of these genes can be seen in Table 3. We identified 99 putative loci for ncRNAs, abbreviated n1 to n99. All above annotated ncRNAs, such as tmRNA (n74) or SRP RNA (n85) were mutually confirmed by our dRNA-seq and *in silico* approaches. Interestingly, known ncRNAs as well as novel ncRNA candidates show a significant level of antisense transcripts (see examples in Figures 10 and 11). For unknown ncRNAs the sense direction is not assignable. Putative ncRNAs, referring to one genomic location and having comparable numbers of cDNA read counts on both strands, are described with the same ID.

**Table 3:** **Selection of highly potential novel ncRNA candidates of** ***A. aeolicus***

**ID**	**Location**			**GC**	**cDNA**	**Annotation**	**Structure and Sequence**					**Remarks**
	**5’ boundary**	**3’ boundary**	**Strand**		**(+/-)**	(**NCBI**/**BacProt**)	**RNALfold**	**Cons_p**	**Cons_t**	**RNAz_p**	**RNAz_t**	
Known ncRNAs												
45	567675	567915	-	0.53	2237/899	murF/UDP	-3.89	11	7	No	0.9990	Downstream of 5S RNA
74	1153499	1153856	-	0.65	83/31	tmRNA/no	-5.04	6	11	No	0.9996	tmRNA
78	1219679	1219903	+	0.55	382/1384	pheT/pheT	-6.59	11	11	No	No	6S RNA
85	1303758	1303875	+	0.57	5239/456	No/no	-4.15	11	11	No	0.7085	SRP RNA
Putative Novel ncRNAs												
2	15301	15474	+	–	0/0	No/no	-3.66	No	5	No	No	Plasmid region
6	69101	69198	-	0.37	809/545	No/no	-4.71	11	11	No	No	
25	328934	328995	+	0.37	582/250	No/no	No	9	9	No	No	
48	620054	620211	-	0.44	41/71	No/no	-3.13	11	9	No	No	
58	739705	739811	+	0.44	41/144	No/no	-3.92	11	11	No	No	
68	989704	989840	+	0.50	476/756	aq_1392/permease	-4.53	4	2	No	No	Aae-65 [85]
74	1153547	1153769	+	0.65	326/51	No/no	-5.04	6	11	No	0.9996	
75	1168974	1169071	-	0.55	158/79	aq_1666/no	-3.84	3	3	No	No	
80	1231909	1232006	+	0.38	860/2339	No/no	-3.74	11	2	No	No	
97	1491199	1491559	-	0.40	10/297	rfaG/glycosyltransferase	-4.60	11	11	No	No	
Tail to tail Transcripts (T2T)												
t2t10	608075	608182	+	0.52	60/20	aq_880/no	-3.70	11	11	No	No	
	608075	608308	-	0.48	22/12	aq_881/DOXP synthase	-3.70	11	11	No	No	
t2t17	1336433	1336708	+	0.46	380/87	aq_1896/predicted	No	11	11	No	No	
	1336544	1336642	-	0.51	100/55	folD/folD	No	11	11	No	No	
t2t20	1479248	1479345	+	0.44	180/117	prmA/prmA	No	11	8	No	No	
	1479168	1479508	-	0.43	12/62	acs’/predicted	-3.19	11	8	No	No	
tRNAs with sense transcripts only												
t06;43	383154	383390	-	0.52	9/2	recN; tRNA/predicted	-3.53	11	10	No	0.9943	
tRNAs with sense and various antisense transcripts												
t34;15	1356464	1356743	+	0.64	23/5	tRNA/no	-5.43	11	10	0.9996	0.9992	
	1356461	1356575	-	0.60	61/15	No/no	-5.43	11	9	0.9996	0.9992	
t44;20	1531016	1531131	+	0.58	1141/437	ihfB/no	-4.33	7	9	No	0.9951	
	1531004	1531130	-	0.56	335/136	tRNA/no	-4.33	7	9	No	0.9951	

The genomic locations and GC-content are listed in columns 2-4. cDNA – the maximal number of observed read counts in the (+)- and (-)-library; Annotation – overlap to predicted proteins by NCBI and BacProt; RNALfold – energy in kcal/mol of locally stable RNA secondary structure predicted by RNALfold; Cons_p and Cons_t – number of species with homologous regions aligned by Pomago and TBA; RNAz – probabilities >0.5 (based on multiple sequence alignments calculated by Pomago (p) or TBA (t)). Further observations, for example that *Aae-65* was described earlier in [85], are noted in the last column. A complete list of novel ncRNA candidates, and tRNAs can be found in the Supplemental Material.

For comparison reasons, we also added tRNAs to our table of ncRNAs, which show the feature of sense-antisense (s/as) expression. To exclude the possibility of mapping or other artefacts, we confirmed the presence of antisense transcripts exemplarily by Northern blots for tmRNA and tRNA 44 (Pro-TGG) (Figure 10).

Furthermore, Northern blots were conducted for the loci encoding candidates n25 and n75, for which the dRNA-seq data indicated sense and antisense transcription each differing between the (+)- and (-)-library (Figure 11). For n25, we found most transcripts on the plus strand in the (+)-library (582), whereas less than half as many transcripts (250) were detected in the (-)-library. Interestingly, an inverse relation was observed for the minus strand (50/361). For n25, Northern blot detection revealed a signal somewhat shorter than the one expected from the cDNA read boundaries, whereas no signal could be detected for antisense transcripts (Figure 11, top). This finding suggests that the sense transcript is the major one. In the case of n75, both sense and antisense transcripts of comparable intensity were detected, the major signals of the Northern blot representing RNAs larger and smaller than anticipated from the read boundaries (Figure 11, bottom). Thus, the polarity of the putative ncRNA gene remains unclear.

Interestingly, very high transcription levels are found in overlapping 5’-upstream regions of two protein-coding genes located on opposing strands (Table 3). Beside these so-called head-to-head (h2h) transcripts we furthermore observed tail-to-tail overlaps (t2t, two 3’-untranslated regions overlapping on opposing strands) that are represented by very high read coverage (Supplemental Material). If these are real transcripts with a certain function or artefacts remains unclear.

## Conclusion

With the advent of a growing number of *Aquificales* genome sequences in public databases, we have re-analyzed this group of bacteria thriving in extreme environments. The *Aquificales* share the feature of a small, compact genome with a reduced number of protein and ncRNA genes. The genes for tRNAs are reduced to a minimum but retain the capacity to decode all types of codons, and rRNA genes are confined to 2–3 copies each. Several classical ncRNAs are present, such as SRP RNA, tmRNA, 6S RNA, RNase P RNA (except for all *Aquificaceae*) and riboswitch candidates in some *Aquificales*. Furthermore, by combining *in silico* analysis with dRNA-seq data of *A. aeolicus*, we were able to predict nearly 100 novel ncRNA candidates, some of which might be specific to the *Aquificales*. Finally, CRISPR systems of bacterial immunity were identified.

Re-annotation of protein genes using BacProt revealed novel proteins with unknown function, some of which might turn out to be specific to the *Aquificales* as well. On average, 63 additional proteins were found that were missing in the respective original annotation.

In our cDNA libraries of *A. aeolicus*, we observed massive amounts of antisense reads with similar patterns (length and amount) at putative ncRNA loci and terminal regions of mRNAs. Examples of transcripts antisense to tmRNA and tRNA are illustrated in Figure 10.

We compared 40 bacterial and 2 archaeal genomes (see Supplemental Material), and the presence or absence of proteins was used to determine their position in the phylogenetic tree of bacteria. Both Archaea form a clear outgroup. *Thermodesulfatator indicus* branches first in the group of Bacteria, followed immediately by the *Aquificales*, while other bacterial branches diverge later. In an additional protein-based analysis, we took the sequences of single-copy orthologs that were present in at least 50% of all species (concatenated 57,260 aa) (see Supplemental Material). In contrast to the protein presence/absence tree, neither the *Aquificales* nor *T. indicus* were placed at a basal position here. However, the two groups are still in close vicinity to each other. This analysis not necessarily excludes the possibility of the *Aquificales* being a basal clade. The selection of orthologs being present in at least 50% of the species leads to a lower coverage of orthologs present in Archaea species and therefore may favor long branch attraction [99]. The idea behind selecting frequently occurring single-copy orthologs was to produce phylogenetic trees being less influenced by horizontal gene transfer. However, proteins shared by Archaea and *Aquificales* only are not part of the selected "50% group" of proteins and are therefore not considered in this analysis.

Both protein-based phylogenetic trees disagree with a previous study [3] where *Desulfobacterium autotrophicum HRM2*, a *δ*-proteobacterium, was added to the *Desulfurobacteriaceae* family based on 16S rRNA analysis. We assume that this was an artefact of the high GC-content of rRNAs due to the high environmental temperatures. Regarding their proteomes, *Aquificales* and *D. autotrophicum* are not significantly related.

The results of the 16S rRNA phylogenetic analysis did not show a clear picture. Depending on the method used for reconstruction, the *Aquificales* were either placed near the root of the bacterial tree (MrBayes and RAxML with GTRGAMMA substitution model) or not (NJ and RAxML with GTRCAT) (see Supplemental Material). In accordance with the results of [26], the *Aquificales* were always placed close to the *Thermotogales* and *Thermales-Deinococcales*, Archaea were more closely related to the *Aquificales* than to the *Thermotogales*.

We identified two 6S RNA and two tmRNA candidate genes in *Hydrogenivirga sp.*, rather than a single one as in the other *Aquificales*. Likewise, *Hydrogenivirga sp.* has a comparatively high amount of tRNA copies and CRISPR loci and its genome is estimated to be of roughly double the size of the other *Aquificales* genomes. Combined, these observations support the notion that the *Hydrogenivirga sp.* genome assembly is erroneous or two genomes of related bacteria (one type from *Hydrogenothermaceae*) have entered the sequencing project, being in agreement with [32]. Based on the tmRNA tag peptides identified in the *Hydrogenivirga sp.* assembly, the second one (*Hydrogenivirga sp.*-B: IPEREIAIAA) matches the sequence exclusively found among the *Hydrogenothermaceae*, although *Hydrogenivirga sp.* belongs to the *Aquificaceae* (see Figure 7). This suggests that the *Hydrogenivirga sp.* assembly is a blend of sequences from a member of the *Aquificaceae* and a member of the *Hydrogenothermaceae*.

## Endnote

^a^Noise cutoff is the highest observed false positive bit score for a potential gene which does not belong to the seed model.

## Competing interests

The authors declare that they have no competing interests.

## Authors’ contributions

Bioinformatical analysis: ML, SW, KR, BMB, NW, and MM. Experimental validation: AIN and RKH. Analyzed data: all. Wrote, read and approved the final manuscript: all.
